# The complete mitochondrial genome of *Ormosia boluoensis*

**DOI:** 10.1080/23802359.2021.1920503

**Published:** 2021-06-28

**Authors:** Zheng-Feng Wang, Yue Zhang, Xiang-Jing Zhong, Ning Kang, Hong-Lin Cao

**Affiliations:** a Key Laboratory of Vegetation Restoration and Management of Degraded Ecosystems, South China Botanical Garden, Chinese Academy of Sciences, Guangzhou, China; b Center for Plant Ecology, Core Botanical Gardens, Chinese Academy of Sciences, Guangzhou, China; c Southern Marine Science and Engineering Guangdong Laboratory (Guangzhou), Guangzhou, China; d Management Bureau of Guangdong Xiangtoushan National Nature Reserve, Huizhou, China

**Keywords:** *Ormosia boluoensis*, mitochondrion, genome assembly, next generation sequencing

## Abstract

*Ormosia* is a particular genus in the Fabaceae family with its striking seeds. The genus *Ormosia boluoensis* is a newly reported and critically endangered species, and field investigations have indicated that there are only hundreds of it. For the effective conservation, we report its complete mitochondrial genome. The length of the *O*. *boluoensis* mitochondrial genome is 248,619 bp, including 28 protein-coding genes, 14 transfer RNA genes, 3 ribosomal RNA genes, and 45 simple sequence repeats. Phylogenetic analysis revealed that *O*. *boluoensis* was a sister to the clade including *Sophora flavescens*, *Ammopiptanthus nanus,* and *Ammopiptanthus mongolicus*.

Fabaceae is the third largest family among the angiosperms, including more than 700 genera and 20,000 species. The *Ormosia* is a monophyletic but small genus in the Fabaceae family. It comprises approximately 130 species (Liu et al. 2019) and China contains 39 of them (http://www.iplant.cn/info/Ormosia). The *Ormosia* species are striking for their seeds, black or red, used as decorations, such as jewelry, beads and other trinkets. *Ormosia boluoensis* Y. Q. Wang & P. Y. Chen is a newly reported species (Wang and Chen 1995). Phylogenetic analysis based on chloroplast genomes indicated that it is genetically close to *O. formosana* (Wang et al. 2020). Unlike some widely distributed congeneric species, *O. boluoensis* is only found in Guangdong Xiangtoushan National Natural Reserve, China, with hundreds of individuals (Guo et al. 2020). It is considered an endangered species. Mitochondria are important intracellular organelles for cellular respiration and metabolism (Mackenzie and McIntosh 1999) and are also useful for taxonomic studies (Duminil and Besnard 2021). Therefore, we sequenced and reported the mitochondrial genome of *O. boluoensis* as a genomic resource for better conservation of this precious species.

Fresh leaves of *O*. *boluoensis* were obtained from the Guangdong Xiangtoushan National Natural Reserve, Huizhou City, China (23°16′44″N, 114°22′26″E). A voucher specimen was deposited at the Herbarium of South China Botanical Garden (Fei-Yan Zeng, zengfeiy@scib.ac.cn) with No. IBSC0000922. The genomic DNA of *O*. *boluoensis* was extracted using a modified CTAB method. The extracted DNA was stored in the Ecological Genetics Laboratory in South China Botanical Garden. The isolated DNA were constructed as a long- and a short-read library. The two libraries were then sequenced using the Nanopore promethION and Illumia HiSeq X Ten platforms. Before *O*. *boluoensis* mitochondrial genome assembly, minimap2 v2.17-r974-dirty (Li 2018) was used to align both the short and long reads to the mitochondrial genomes in Fabaceae (Figure 1) and the mapped reads were then extracted. Flye 2.8.1-b1676 (Kolmogorov et al. 2019) was subsequently used to *de novo* assemble the *O*. *boluoensis* mitochondrial genome with the extracted long reads. After assembly, the mitochondrial genome was polished by Racon v1.4.13 (Vaser et al. 2017) using the long reads and nextPolish v1.2.3 (Hu et al. 2020) using the short reads, respectively, each with two runs. The polished genome was annotated with GeSeq (Tillich et al. 2017). The simple sequence repeat (SSR) was identified with MISA-web (Beier et al. 2017). After annotation, the assembled *O*. *boluoensis* mitochondrial genome and its annotated files were submitted to GenBank (the accession number MW455117). To perform phylogenetic analysis for *O*. *boluoensis*, the mitochondrial genomes of the other 18 species including 16 Fabaceae species and two outgroup species (*Nicotiana attenuate* and *Nicotiana tabacum*) were downloaded from GenBank. 20 shared protein coding genes in their mitochondrion genomes were then extracted, translated into amino acid sequence, aligned and concatenated using PhyloSuite 1.2.2 (Zhang et al. 2020). IQ-TREE 2.1.2 (Nguyen et al. 2015) was used to perform maximum likelihood phylogenetic inference and the JTTDCMut model was selected as best-fit model by ModelFinder (Kalyaanamoorthy et al. 2017).

**Figure 1.**
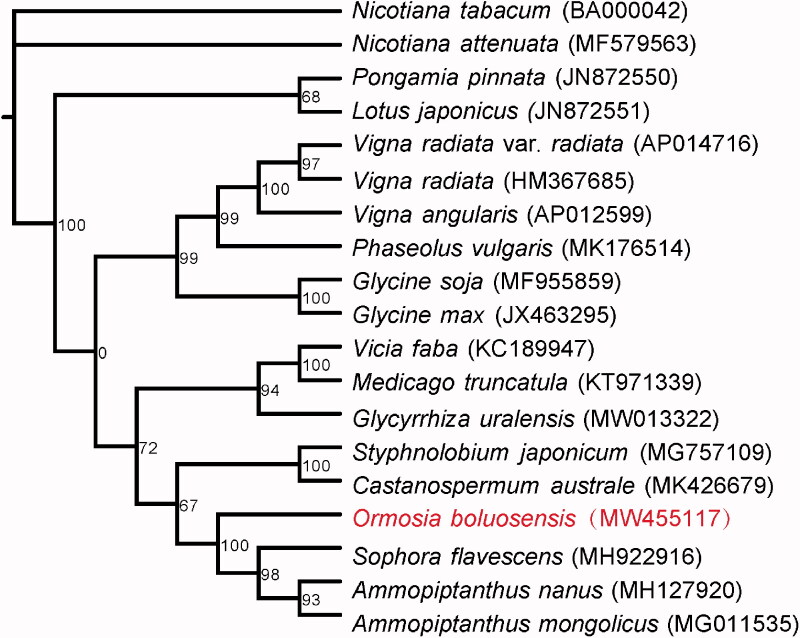
Phylogenetic tree for *Ormosia boluoensis* and the other species using their complete mitochondrial genomes. The GenBank accession numbers of species are shown in parentheses. Bootstrap support values in % are shown at nodes.

The mitochondrial genome of *O*. *boluoensis* was 248,619 bp in length. Its GC content was 45.22%. The mitochondrial genome included 28 protein-coding genes, 14 transfer RNA genes, and 3 ribosomal RNA genes. SSR analysis indicated that *O*. *boluoensis* mitochondrial genome contained 45 SSRs (Table S1), in which mononucleotide-repeat SSRs were the most abundant with 25 SSRs, followed by pentanucleotide-repeat SSRs with 11 SSRs. Phylogenetic analysis supported a monophyletic relationship of *O*. *boluoensis* to the clade of *Sophora flavescens*, *Ammopiptanthus nanus,* and *Ammopiptanthus mongolicus* (Figure 1).

## Data Availability

The complete mitochondrial genome sequences of *Ormosia boluoensis* have been deposited in GenBank under the accession number MW455117 and is also accessible at https://doi.org/10.13140/RG.2.2.26491.95523. The associated BioProject and Bio-Sample numbers are PRJNA689914, SAMN17221877, SRA for short reads and long reads are SRR13364204 and SRR13364203 respectively.
